# Evaluation of the use of Swedish integrated electronic health records and register health care data as support clinical trials in severe asthma: the PACEHR study

**DOI:** 10.1186/s12931-016-0461-1

**Published:** 2016-11-15

**Authors:** Stefan Franzén, Christer Janson, Kjell Larsson, Max Petzold, Urban Olsson, Gunnar Magnusson, Gunilla Telg, Gene Colice, Gunnar Johansson, Mats Sundgren

**Affiliations:** 1 Register Centrum, Gothenburg, Sweden; 2 Department of Medical Sciences, Respiratory, Allergy and Sleep Research, Uppsala University, Uppsala, Sweden; 3 Department of Environmental Medicine, Karolinska Institute, Solna, Sweden; 4 Centre for Applied Biostatistics, University Gothenburg, Gothenburg, Sweden; 5 Statisticon AB, Uppsala, Sweden; 6 AstraZeneca, Gothenburg, Sweden; 7 AstraZeneca Nordic-Baltic, Södertälje, Sweden; 8 AstraZeneca, Gaithersburg, USA; 9 Public Health and Caring Science, Family Medicine and Preventive Medicine, Uppsala University, Uppsala, Sweden; 10 Biometrics and Information Science, AstraZeneca, Gothenburg, SE-431 83 Mölndal Sweden

**Keywords:** Electronic health records, Registries, Asthma, Exacerbation, Placebo studies

## Abstract

**Background:**

In the development of new drugs for severe asthma, it is a challenge from an ethical point of view to randomize severe asthma patients to placebo, and to obtain long-term safety data due to discontinuations. The aim of this study was to evaluate the feasibility of using electronic health record (EHR) data to create a real-world reference population of uncontrolled asthmatic patients to supplement the concurrent control/placebo group in long-term studies of asthma.

**Methods:**

EHR data from 36 primary care centres and a University hospital in Sweden were linked to Swedish mandatory health registers (2005–2013), creating a population covering 33 890 asthma patients, including data on co-morbidities, risk factors and laboratory/respiratory measurements. A severe asthma EHR reference cohort was established. We used logistic regression to estimate the propensity score (probability) of each RCT or EHR patient existing in the EHR cohort given their covariates.

**Results:**

We created an EHR-derived reference cohort of 240 patients, matching the placebo group (N = 151) in an RCT of severe asthma. The exacerbation rate during follow-up in the EHR study population was 1.24 (weighted) compared to 0.9 in the RCT placebo group. Patients in the EHR cohort were of similar age as in the RCT placebo group, 50.6 years versus 50.1 years; had slightly higher body mass index 27.0 kg/m^2^ versus 27.3 kg/m^2^; and consisted of 40% versus 34% males.

**Conclusions:**

The results indicate that EHRs provide an opportunity to supplement the control group in RCTs of severe diseases.

## Background

Asthma is a common but complex heterogeneous chronic inflammatory disease of the airways, which presents with variable symptoms of cough, breathlessness, and wheeze, with episodic acute worsening of symptoms known as exacerbations, particularly in severe asthma [1]. The prevalence of asthma in Sweden is 8-10% [2] and pharmacological treatment to relieve symptoms and maintain optimal lung function is required, but despite various treatment options, a large proportion of patients have asthma that remains uncontrolled [3]. These patients have an increased risk of developing severe exacerbations, suffer from a poor quality of life, and pose a high economic healthcare burden [3].

There is a broad spectrum of novel therapeutic agents currently under development for the treatment of severe asthma. Development of a new drug requires extensive documentation of effectiveness and safety, usually by comparison with a placebo control group in a RCT. When the indication involves severe asthma, it may be an ethical challenge to randomize patients to a long-term safety follow-up in a control group receiving placebo [4]. Difficulties enrolling severe asthma patients to long-term placebo controlled trials may have a negative impact on the development time of new innovative drugs, a disadvantage to patients in need [5]. Moreover, it may be difficult keeping severe asthma patients in the placebo arm of a long-term safety study data due to discontinuations [6]. Implications include for example the inability to contextualise any findings in the active control arm.

In parallel, clinical research is on the threshold of a new era in which electronic health records (EHR) are gaining an important novel supporting role [7]. The transition into EHRs has been far from uniform in different parts of the world and has not mirrored general information communication technology (ICT) developments. In some regions, including Scandinavia and the UK, electronic systems were first adopted by primary care, whereas in others, the development was led by university clinics in large hospitals [8]. Whilst EHRs used for routine clinical care have some limitations at present, new improved systems and emerging research infrastructures are being developed to ensure that EHRs can be used for secondary purposes such as clinical research, including the design and execution of clinical trials for new medicines [9]. Still, given the poor quality of many legacy EHR systems, it is not surprising that their use for clinical research has been limited. In many cases, special disease registers at a regional or national level (often termed quality registers) have been created with special reporting outside the normal clinical record, primarily to support quality improvement in health care systems but also to serve research purposes. Some countries have invested substantially in such registries; for example, Sweden’s ‘national quality registers’ include more than 100 disease conditions on a national scale and collect high quality data with coverage that may be near 100% of all cases for some of these conditions [10]. This has created much valuable data, many international publications and a significant impact on the practice of medicine. However, the registry structures are inflexible and create significant work, even if EHR extracts using modern standards can partially automate registry data capture, as has been demonstrated for the Swedish Heart Failure Register and the Swedish National Diabetes Register [11].

Another common health data source for clinical research, especially in the US, is health insurance administrative data, or claims data. Claims data include diagnostic information, treatments given, and providers used, in addition to a variable number of financial measures [12]. This makes them appealing to researchers as they offer numerous advantages such as anonymous, plentiful, inexpensive, and widely available information in electronic format. However, because these data systems were developed for administrative purposes they often lack quality and adequate measures needed for clinical research. Moreover, claims data in many countries are limited to patients that can afford health insurance thereby creating a socio-economic bias [13].

Currently, we see an increasing pace of implementation of modern quality-controlled EHRs with growing evidence that EHRs can support clinical research including but certainly not limited to clinical trials for new medicines (e.g., optimizing and validating clinical protocols and supporting identification of investigator hospital sites) [7]. To further explore the value of EHR data for clinical research it is important to evaluate to what extent EHR systems have reached the maturity to provide more integrated support to randomized clinical trials (RCT). One such example is the use of EHR data to create a real-world reference population of patients to supplement the control/placebo group in long-term studies of severe diseases.

The aim of this propensity score weighted, retrospective, observational cohort study was to evaluate the feasibility of creating an EHR/registry-based reference cohort comparable to the placebo group of a previously completed randomized clinical trial (RCT) in severe asthma.

## Methods

### Study design, protocol and data sources

This was a propensity score weighted, retrospective study where the placebo group population and its outcome in an RCT was compared to an observational cohort, linking data from national mandatory Swedish registries to primary care EHR data.

The Swedish National Board of Health and Welfare performed data linkage and the Department of Medical Sciences, Respiratory Medicine at Uppsala University, Sweden managed the linked database.

Thirty-six centres and one university hospital were included, with a catchment area covering 4% of the Swedish population. No stratification of primary healthcare centres was performed, but effort was made to reflect Swedish asthma healthcare by selection of centres that covered a representative sample of the Swedish asthma population, by a mix of rural and urban areas, public and private providers, and centre size [14]. EHR data (e.g., date of birth, gender, diagnoses by ICD-10 codes, number of primary healthcare centre contacts, lung function assessments, prescribed medications, exacerbations, and laboratory variables) were extracted using an established software system (Pygargus Customized eXtraction0, Program, CXP™) [15].

Data were also extracted retrospectively from Swedish national registers, covering mandatory individual health data on a full population level. Data regarding morbidity and mortality were collected from the National Patient Register, including inpatient hospital care (admission and discharge dates, main and secondary diagnoses specified by ICD-10 code) and outpatient hospital care (number of contacts, diagnoses), and the Cause of Death register, including date and cause[s] of death, respectively. Data on drug prescriptions from hospital and primary care were collected from the Swedish Prescribed Drug Register. We were able to follow patients for up to nine years (2005-2013). The regional ethics committee in Uppsala, Sweden approved the study protocol.

**Fig. 1:**
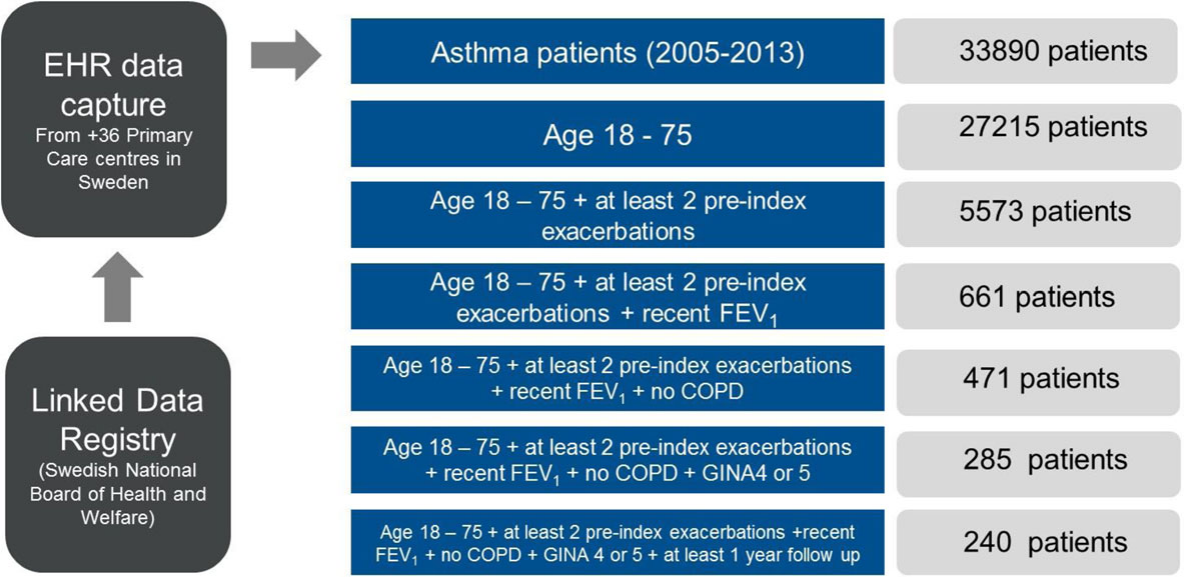
Data flow for creating the EHR placebo population

The base population (primary data set) included males and females, with a record of drug prescription for obstructive pulmonary diseases (Anatomical Therapeutic Chemical (ATC) code R03) and/or a physician-diagnosed asthma (ICD-10 code J45-J46) in the primary care setting. No exclusion criteria were predefined. To preserve patient anonymity the social security number used to identify patients was replaced with a study ID number prior to further data processing. Selection of patients from the EHR base population into the EHR study cohort consisted of a two-step approach: We first extracted data from EHRs (*n* = 33890) and sent it to the Swedish National Board of Health and Welfare, which linked this database to the pre-defined national registers (e.g., patient, prescription of drug, and cause of death registries) [16] (Fig. 1). We then needed to identify patients (*n* = 240) eligible for inclusion in a predefined RCT study using the RCT inclusion and exclusion criteria.

### RCT study population

To compare to the EHR study cohort, a data set of de-identified RCT placebo data was derived from a completed AstraZeneca-sponsored randomised, double-blind, placebo-controlled, parallel-group, multicentre, phase 2b study in severe asthma [17]. A total of 452 patients were randomly assigned (1:1) to one of two dosing regimen groups and further randomised (2:1) to receive tralokinumab or placebo. For the current PACEHR study, the entire RCT population (*n* = 452) is used to represent the placebo group for baseline (index date) comparisons; since the study is randomized, they represent a larger sample of the same patients as the placebo group at baseline. For outcome assessments, the RCT placebo data set used consisted of results from the 151 placebo patients in this study.

The RCT study design consisted of a 5 week screening and run-in period, a 48 or 50 week treatment period (depending on dosing regimen), and a 22 week safety follow-up period. Enrolled patients were aged 18–75 years with severe uncontrolled asthma, consistent with the European Respiratory Society and American Thoracic Society definition [18]. Patients were receiving high dose inhaled corticosteroids (total daily dose >500 μg fluticasone dry powder inhaler or equivalent via metered dose inhaler) plus a long acting beta agonist (LABA) at least 30 days before visit 1, and had at least two, but no more than six, exacerbations in the previous 12 months. The primary outcome variable was the annual asthma exacerbation rate at week 52. Additional secondary outcomes included lung function endpoints, Asthma Control Questionnaire-6 and Asthma Quality of Life Questionnaire at week 52 [17].

### Outcome variables

**Fig. 2:**
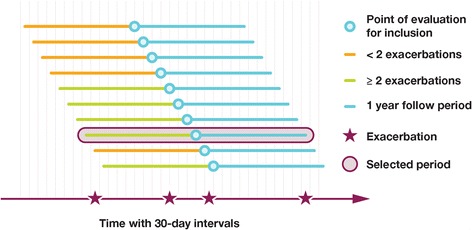
Index date methodology for randomising the EHR population

For this study, the definitions for outcome variables were the same as those used in the RCT study. Asthma exacerbation was defined according to established criteria, which consisted of an increase in asthma symptoms resulting in use or increase in dose of systemic corticosteroids for three or more consecutive days [18]. The number of exacerbations was evaluated during a 12-month follow up period starting from the index date in the EHR study cohort and from the date of randomization in the RCT study population (Fig. 2). Lung function was determined by spirometry and expressed as the percent of predicted normal forced expiratory volume in one-second (FEV_1_).

### Statistical analysis

Conceptually, the patients included in the RCT were assumed to constitute a sample from a population of eligible patients, where the actual sampling probability is unknown and varies between patients depending on patient characteristics. This probability can be operationalized as a propensity score [19, 20], although in the current analysis, the compared study populations are defined by inclusion or not in an RCT, as opposed to being exposed or not to a certain treatment. As the aim was to use the patients in the EHR population as a control (comparison) group, the estimated outcomes in the EHR population were adjusted for the selection probabilities derived from the propensity score model. Specifically, to accomplish this we weighted the outcome estimate with the inverse of the probability for each EHR patient of being an RCT patient [21, 22]. We used a logistic regression model on the combined dataset of RCT placebo patients and EHR reference cohort patients, with inclusion in RCT yes/no as outcome, to estimate the selection probabilities, and including age, gender, BMI, GINA classification, FEV_1_ and the number of pre-index exacerbations as independent variables [23].

We used a weighted Poisson regression model including age to estimate the exacerbation rate. To estimate the average FEV_1_ we used the arithmetic mean at each visit in the RCT and both unweighted and weighted arithmetic means during the whole 12-month follow up period in the EHR population. Confidence intervals for the weighted average, considering weights, were determined using robust standard errors. The analysis was done using SAS (V.9.4, SAS Institute Inc. North Carolina, USA) and R for Windows (V. 3.2.3, R Foundation) statistical software.

For the selection of EHR patients, we evaluated each patient with respect to inclusion criteria in the RCT at regular time points 30 days apart. At each of these time points, we considered the patient age and number of exacerbations for the 12 months prior to that time point. A patient was considered eligible at the time point if the age was between 18 and 75 and the patient had two or more pre-index exacerbations. If the patient were eligible at more than one time point, one of the time points was randomly selected as the index date.

## Results

Patients in the EHR study cohort were of similar age as the patients in the RCT population, mean (SD) 50.3 (14.8) years versus 50.1 (12.3) years, had slightly higher body mass index (BMI), 28.0 (5.8) kg/m^2^ versus 27.3 (4.9) kg/m^2^, and consisted of 31.7% versus 34.4% males. The patients in the EHR study cohort were more likely to be in GINA 5, 42.1% compared to 17.1% in the RCT study population and had on average 3.0 exacerbations during the 12 months preceding the index date compared to 2.6 in the RCT population. The mean FEV_1_ percentage was 84.1% in the EHR study cohort and 68.7% in the RCT population. Details on comorbidities, medications used at baseline index date and laboratory variables for the EHR population are available in the appendix.

Applying weights increased the similarity between the RCT and the EHR patients on baseline characteristics, with the most prominent effect on pre-bronchodilator FEV_1_ (% predicted) and the proportion of GINA 5 patients. Results show for the EHR population a weighted average (SD) pre-bronchodilator FEV_1_ (% predicted) of 63.1 (31.8), compared to the unweighted average 84.1 (19.0) and in the proportion of GINA 5 patients a weighted percentage of 13.3% against the unweighted 42.1% (Table 1).

**Table 1:** Descriptive statistics on weighted vs. un-weighted confounders

Variable	EHR (unweighted)		EHR (weighted)		RCT total population	
	*n*	Mean (SD %)	*n*	Mean (SD %)	*N*	Mean (SD %)
Age	240	50.3 (14.8)	239	50.6 (23.7)	451	50.1 (12.3)
BMI (kg/m^2^)	239	28.0 (5.8)	239	27.0 (7.6)	451	27.3 (4.9)
FEV (% predicted)	240	84.1 (19.0)	239	63.1 (31.8)	450	68.7 (18.0)
GINA 5 n (%)	240	101.0 (42.1%)	239	70.0 (13.3%)	451	77.0 (17.1%)
Male n (%)	240	76.0 (31.7%)	239	106.1 (40.1%)	451	155.0 (34.4%)
Neutrophils (unit)	77	6.2(2.8)	77	6.0 (2.8)	426	4.7 (2.0)
One year Pre-index Exacerbations rate	240	3.0 (1.6)	239	2.5 (1.5)	451	2.6 (0.9)

Weighted and unweighted patient characteristics of the EHR population compared to the combined placebo and treatment groups in RCT

**Fig. 3:**
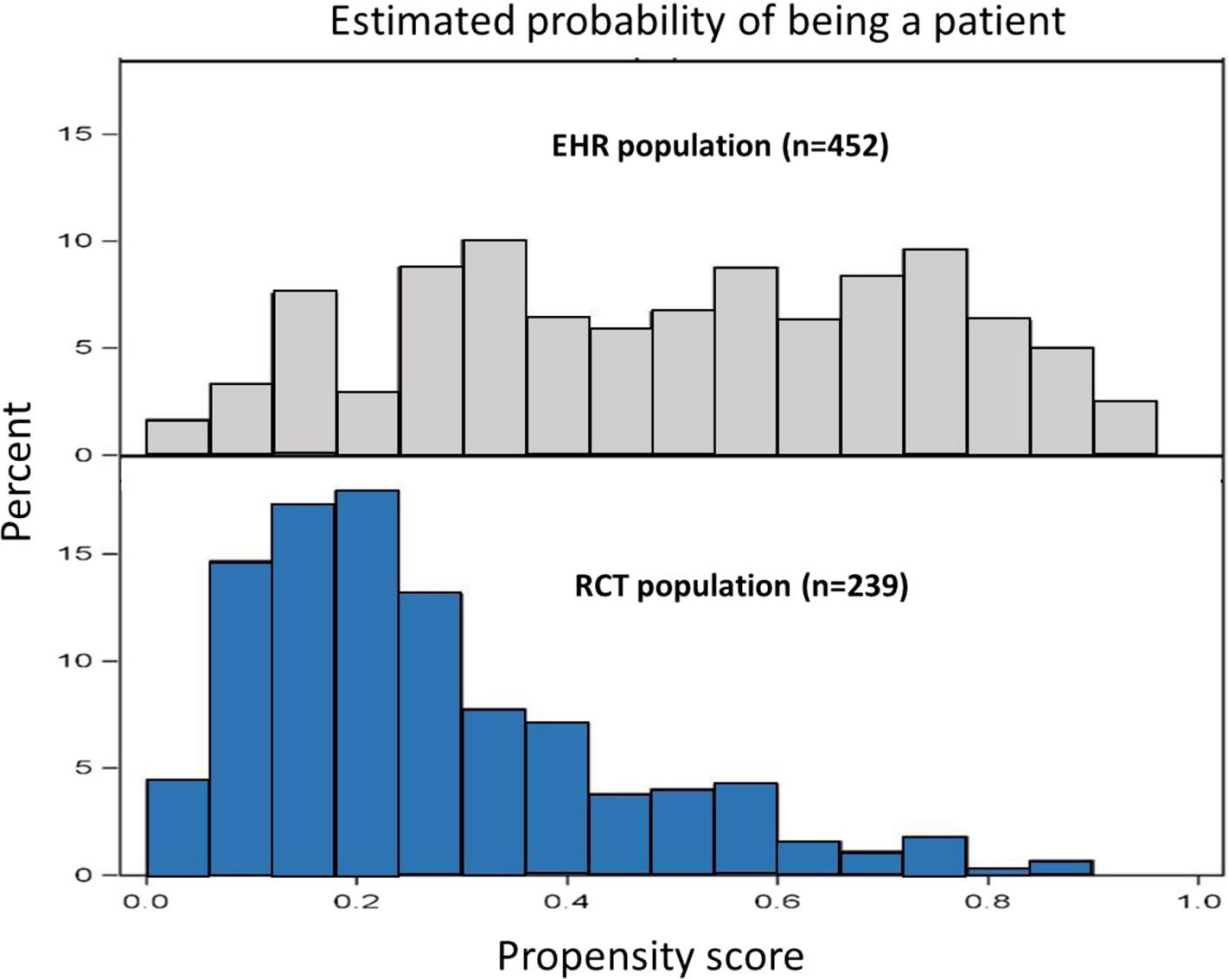
Distribution of the estimated probability of being EHR patient vs the RCT population

The distribution of the estimated propensity scores indicated that the unweighted EHR population and the RCT population are not directly comparable (Fig. 3) although the main assumption of no patients having propensity scores of zero or one is fulfilled (Table 2).

**Table 2:** Descriptive statistics of the estimated propensity scores of EHR and RCT data

Population	n	Mean	SD	Min	Median	Max
RCT	239	0.50	0.24	0.03	0.51	0.95
EHR	450	0.26	0.17	0.03	0.23	0.88

The one year exacerbation rate in the placebo group of the RCT was 0.9 [95% CI 0.76, 1.08] and 1.86 [95% CI 1.70, 2.05] in the EHR study population. The weighted exacerbation rate in the EHR study population becomes 1.24 [95% CI 0.94, 1.63], which is substantially closer to the exacerbation rate in the placebo group in the RCT (Table 3).

**Table 3:** Estimated exacerbation rates of RCT and EHR data

Population	Analysis	Rate	Lower 95% CI	Upper 95% CI
RCT (placebo)	Unweighted	0.90	0.76	1.08
EHR	Weighted	1.24	0.94	1.63
EHR	Unweighted	1.86	1.70	2.05

Weighted and unweighted exacerbation rates for the EHR population (*n* = 240) and unweighted exacerbation rate for placebo arm in the RCT (*n* = 151)

The patients in the RCT had spirometry performed at each visit. The average FEV_1_ ranged from 68.3 [65.7, 71.0] (mean with 95% CI) at the baseline visit to 71.5 [68.6, 74.4] (mean with 95% CI) at visit 13 at 52 weeks. Only 74 EHR patients had spirometry performed during the 1-year post-index period. The average FEV_1_ for these patients was 78.2 [72.7, 83.7] (mean with 95%CI). The corresponding weighted average with 95% CI was 68.0 [58.3, 77.8] which again is much closer to what is observed in the placebo group of the RCT.

In the RCT population, FEV_1_ was observed under controlled conditions at scheduled visits leading to a large number of repeated observations per patient. In the EHR population FEV_1_ was observed as part of routine spirometry as needed by the treating physician and hence not all patients in the EHR population have any observations on FEV_1_ during the 12-months post index period and those who do have one or perhaps two observations (Appendix 1).

## Discussion

In the present retrospective, observational cohort study based on a population of 33 890 asthma patients, with up to nine years of follow-up in Sweden, we showed that it is technically feasible to create a reference cohort (*N* = 240) which has reasonable similarity to an RCT placebo group (*N* = 151) in severe asthma. This was accomplished both by finding an adequate number of patients and by creating high data quality (i.e., capturing the key inclusion/exclusion criteria to ensure reasonable comparability). We used asthma exacerbation frequency, which also was the primary outcome in the RCT study, as a key indicator to evaluate the comparability achieved between the RCT and EHR placebo groups.

Previous research has been successful to create high quality data from registries in the area of asthma [24]. To our knowledge, this study is the first to examine the possibility to mimic a randomized placebo RCT cohort based on real world data. A major strength is that this study is based on a large population sample in a well-defined geographical area. The weighted analysis approach proved successful to largely account for differences in sampling probability between the RCT and the EHR patients, as assessed by comparable estimates of exacerbation rate and FEV_1_. The uniqueness of this study is the actual successful extraction, and formation, of a de-identified data set, linking Swedish primary care medical records data with a number of Swedish National health registers, and then to provide a pool of patients which mimic the characteristics of a RCT placebo group in severe asthma. The high quality of the registry data allowed us to find the required data elements.

Creating an EHR population that is analogous to a RCT placebo population has several difficulties. First, we had to operationalize the inclusion criteria based on data that are routinely captured in EHR, as opposed to what is captured in a Case Report Form (CRF) based on a study protocol. There can be substantial differences in the timing of assessments, as well as in the actual measurements used. Furthermore, patients participating in a RCT are not in fact as assumed by our method a true random sample from the population found in an EHR source. While between subject differences in sampling probability due to observed variables might be adjusted for, as was done in this analysis, there can easily be differences due to unobserved variables. Having access to detailed information on diseases and medications pre index from the trial for the RCT patients and from in-hospital and pharmacy claims registries will increase the likelihood of successfully adjusting for differences in selection probabilities related to disease severity and co existing conditions. Other factors such socioeconomic factors are much harder to account for. While there are registries containing socioeconomic data such as income, education and ethnicity in terms of country of origin available in some countries (e.g., Sweden) these data would rarely be available for the patients in the RCT even if the study was done in such a country, mainly depending on the strict rules of data integrity surrounding an RCT.

Lastly, the way that outcome measures are observed needs to be consistent for the EHR and RCT populations. There are commonly accepted standards for performing spirometry, but this test is performed less consistently in regular practice than in an RCT. Consequently, observations on FEV_1_ in the EHR cohort may differ from those obtained in the RCT in timing and frequency. Exacerbations are partly defined by use of OCS, and the distinction between continuous use and acute use may not be completely clear in registry data while it is unambiguously recorded in the case report form for an RCT.

Questionnaire approaches to assessing patient reported outcomes are commonly used in RCTs but infrequently are included in EHRs. Issues like these might lead to systematic differences in the observed outcome that are impossible to statistically account for and for future studies hard clinical endpoints such as death of other well defined events may be better choice if relevant.

The use of actual EHR data linked to various sources, prescription data and laboratory data including spirometry data, presents a unique opportunity to create a dataset that is sufficiently rich to address the difficulties mentioned above. Both of the two most important confounders, the number of exacerbations during the 12 months preceding the index date and the pre index FEV_1_, were captured for a sufficiently large number of patients, similar to the RCT sample size. However, some laboratory variables were sparsely collected in the EHR population and there were difficulties in finding relevant observations on some of the laboratory variables key to the RCT, such as neutrophils and eosinophils. This mainly reflects that these variables only rarely are used in general clinical practice. Further details of captured data variables in the EHR reference cohort can be found in Appendix 2.

### Limitations

There are several important assumptions that underpin the analysis. Firstly, the inclusion and exclusion criteria need to be equivalent. In the RCT placebo cohort, we had the opportunity to assess pre-randomization variables according to a specific protocol. In the EHR cohort, the corresponding criteria had to be operationalized based on routinely available observations recorded in the EHR. Therefore, the inclusion criteria may not be equivalent, which may lead to systematic differences in the RCT and EHR population.

Secondly, we assumed that the RCT study population was a random sample conditional on the patient characteristics used to estimate the weights that we used to create the EHR reference. This is equivalent to the assumption of “*no unobserved*” confounders commonly used in epidemiology. Although capturing data on the most obvious confounding variables, such as the number of pre-index exacerbations, pre index GINA classification and spirometry, we cannot be certain that there are no unobserved confounders. The analysis technique used here is also not able to adjust for the notion of a placebo effect arising from the very participation in an RCT.

Furthermore, we must assume that the outcome measure is recorded in the same way in the RCT and EHR population. Exacerbations are partly defined as increased acute use of OCS, which is difficult to distinguish from continuous use based on the drug dispense registry. Lastly, the results for the RCT placebo cohort used in this study were taken from a large multicentre trial with patients from 16 countries not including Sweden. The EHR data used comes from primary care centres in Sweden. Asthma care in most countries generally follows international guidelines [23] but asthma prevalence [25], as well as the placebo exacerbation rate in trials, are known to vary among countries [26]. These observations may make it difficult to relate a global RCT to a population in a single country that was in this case not even represented in the trial.

Another potential limitation when using EHR data for creating a reference group is the risk of overestimating the treatment effect. This is due to the fact that in RCTs there is often an improvement in the control groups because the patients have improved asthma care due to regular visits. Thus the effect in the treatment group is combination of the improved asthma care and the drug treatment. This limitation is supported by the fact that the prevalence of exacerbations was higher in the EHR groups than the RCT control group.

### Further research and opportunities

This study demonstrates the feasibility of capturing high quality health data in EHRs and offers the possibility of creating a real-world reference population of uncontrolled severe asthma patients to supplement or even potentially replace the control/placebo group in long-term studies. Results provide evidence for the value of EHR data to improve the design and interpretation of clinical trials. Moreover, our results support the opportunities to conduct registry-based trials or so-called mixed trials where a placebo cohort can prospectively go alongside a RCT study especially in disease areas where it is problematic to assign patients to placebo treatment. For instance long term extension studies are presently often uncontrolled one-armed designs without an internal reference. One concern with this study design is unanticipated adverse events. Adding an external reference cohort based on registry data using the technique presented in this paper may aid the interpretation of such a finding in this type of study. Further research should validate the methodology within other disease areas. For the future, the PACEHR approach would render important value like for example long term safety study for asthma in which the patient is receiving a biologic agent in development, a control group is simply not be feasible, or for other diseases like atopic dermatitis. For both these cases, among others, identifying an appropriate control group from EHRs to be followed prospectively would be perfectly suitable and helpful.

## Conclusion

This study demonstrate that an adequate large number of severe uncontrolled asthma patients, comparable to the placebo group in a RCT, can be found in Swedish EHRs based on pre-defined criteria for uncontrolled asthma. Causal inference methodology based on propensity score analysis and weighting methods adjusted for differences in observed confounders, and can provide a useful tool to aid the interpretation of RCTs. The results indicate that EHRs provide an opportunity to supplement, the control group in RCTs of severe diseases.

## Appendix 1

**Table 4:** EHR spirometry data (average FEV1) from RCT & EHR cohorts

RCT data per visit #	N	Mean	Lower	Upper
1	151	68.3	65.7	71.0
3	150	70.2	67.4	73.0
5	145	69.9	67.0	72.9
9	143	69.9	66.9	73.0
13	142	71.5	68.6	74.4
17	137	69.9	66.8	73.0
21	132	70.2	67.0	73.3
25	134	69.4	66.3	72.5
33	129	69.5	66.3	72.7
41	129	70.2	67.0	73.3
49	130	69.4	66.1	72.7
53	129	69.1	65.9	72.3
59	127	70.4	67.4	73.5
67	111	70.4	67.1	73.7
75	77	70.4	65.8	74.9
EHR data	N	Mean	Lower	Upper
Unweighted	74	78.2	72.7	83.7
Weighted	73	68.0	58.3	77.8

## Appendix 2

**Table 5:** EHR cohort (*N* = 367): Comorbidity at index

Condition	*N* (%)
Respiratory-related conditions	118 (49.2)
Acute upper respiratory tract infections	161 (67.1)
Chronic bronchitis	11 (4.6)
COPD	NA
Asthma	NA
Pneumonia and influenza	59 (24.6)
Influenza	4 (1.7)
Pneumonia	58 (24.2)
Other respiratory system diseases	4 (1.7)
Other diseases of the upper respiratory tract	83 (34.6)
Non-allergic	14 (5.8)
Allergic	50 (20.8)
Chronic rhinitis	14 (5.8)
Other relevant conditions	124 (51.7)
Diabetes	13 (5.4)
Type 1	3 (1.3)
Type 2	10 (4.2)
Metabolic Disorders	26 (10.8)
Hypertensive diseases	56 (23.3)
Ischaemic heart disease	8 (3.3)
Unstable angina	NA
Angina pectoris	3 (1.3)
Myocardial infarction	5 (2.1)
Chronic sinusitis	7 (2.9)
Heart failure	1 (0.4)
Malignant neoplasm	4 (1.7)
Cerebrovascular diseases	0 (0.0)
Haemorrhage	1 (0.4)
Cerebral infarction / Stroke	1 (0.4)
Rheumatoid arthritis	NA
Polymyalgia rheumatic	56 (23.3)
Anxiety and Depression disorder	28 (11.7)
Anxiety	46 (19.2)
Depression	8 (3.3)
Nasal polyps	21 (8.8)
Osteoporosis/Fractures	6 (2.5)
Osteoporosis	NA
Eczema	14 (5.8)
Inflammatory bowel disease	6 (2.5)
Crohn's disease	3 (1.3)
Other acute lower respiratory infections	97 (40.4)
Chronic lower respiratory diseases	4 (1.7)

NA = value not found or zero

**Table 6:** EHR cohort (*n* = 367): Concomitant medication at index

Treatment	*n* (%)
ASTHMA MEDICATIONS	367 (100.0%)
Inhaled corticosteroids (ICS)	215 (58.6)
Long-acting ß2-agonists (LABA)	124 (33.8)
ICS/LABA combination	208 (56.7)
Adrenergic in combination with anticholinergics	5 (1.4)
Short acting	5 (1.4)
Long acting	NA
Leukotriene modifiers (LTRA)	113 (30.8)
Methylxanthines	4 (1.1)
Anti-IgE treatment	NA
Short-acting ß2-agonists (SABA)	256 (69.8)
Systemic corticosteroids (OCS)	367 (100.0)
Anticholinergics	21 (5.7)
- Short acting	9 (2.5)
- Long acting	13 (3.5)
Omalizumab	NA
PD4 antagonists	NA
N-acetylcysteine	126 (34.3)
Cardiovascular medications (ANY)	155 (42.2)
Anti-dyslipidaemias	50 (13.6)
Statins	48 (13.1)
Fibrates	1 (0.3)
Anti-hypertensives	90 (24.5)
Beta-blockers	47 (12.8)
ACEs	29 (7.9)
ARBs	44 (12.0)
Other medications	111 (30.2)
Antibiotics	169 (46.0)
Anti-virus	11(3.0)
Anti-allergy	3 (0.8)
Cough and cold	223 (60.8)
Antihistamines	201 (54.8)
Nasal corticosteroids	162 (44.1)
Antidepressant	65 (17.7)
Hypnotics	46 (12.5)
Antianxiety	63 (17.2)
Bifosfonates	10 (2.7)
Calcium D vitamine	2 (0.5)

NA = value not found or zero

**Table 7:** EHR cohort (n = 367): Lab test values at index

Treatment	*n* (%)	Mean(SD)
ALAT	72	0.5 (0.3)
Blood glucose	114	6.1 (1.7)
LDH	37	3.3 (0.9)
HDL	38	1.5 (0.5)
LDL/HDL	37	2.3 (0.8)
Total Cholesterol	43	5.5 (1.2)
Triglycerides	38	1.6 (0.7)
Thrombocytes	143	300.7(81.4)
Eosinophils	33	0.2 (0.1)
Leukocytes	147	8.4 (3.2)
Neutrophils	77	6.2 (2.8)
ALP	60	1.3 (0.3)
ASAT	31	0.5 (0.3)
Albumin	28	36.6 (3.1)
Haematocrit	143	40.9 (3.6)
Haemoglobin	163	138.9 (12.2)
Potassium	114	3.9 (0.4)
Sodium	101	139.5 (2.5)
Creatinine	62	66.7 (12.9)

## Abbreviations

ATC code: Anatomical therapeutic chemical code
BMI: Body Mass Index
EHR: Electronic health records
FEV_1_: Percent of predicted normal Forced Expiratory Volume in one-second
GINA: Global initiative for asthma guidelines
ICD-10 code: International statistical Classification of Diseases and related health problems 10th Revision
LABA: Long acting beta agonist
RCT: Randomised clinical trials
SAS: Statistical analysis system software
